# Guanfacine as a potential preventive therapy for chronic migraine: a mechanistic hypothesis

**DOI:** 10.3389/fneur.2026.1947051

**Published:** 2026-09-17

**Authors:** Tigran Kesayan, Karan Patel

**Affiliations:** 1 Department of Anesthesiology, Division of Pain Medicine, Vanderbilt University Medical Center, Nashville, TN, United States; 2 Department of Neurology, Vanderbilt University Medical Center, Nashville, TN, United States; 3 Department of Plastic Surgery, Vanderbilt University Medical Center, Nashville, TN, United States

**Keywords:** ADRA2B, central sensitization, CGRP, chronic migraine, drug repurposing, guanfacine, headache, migraine prevention

## Abstract

**Background:**

Despite the recent therapeutic advances, approximately one-third of patients with chronic migraine fail to achieve clinically meaningful benefit, and existing oral preventive therapies remain limited by tolerability and off-target effects. There is, therefore, a pressing need for new, well-tolerated, mechanistically distinct preventive treatments for chronic migraine. This hypothesis article evaluates the pharmacological and biological rationale supporting guanfacine as a potential preventive therapy for chronic migraine.

**Hypothesis:**

We hypothesize that guanfacine, a selective α2A-adrenergic receptor agonist already approved for hypertension and attention-deficit/hyperactivity disorder, may have the potential to prevent chronic migraine through three biologically plausible convergent mechanisms: (1) potential presynaptic inhibition of calcium-dependent release of CGRP, glutamate, substance P, and other pro-nociceptive neuropeptides from trigeminal primary afferents; (2) potential attenuation of peripheral and central sensitization within the trigeminovascular system and the trigeminocervical complex; and (3) potential modulation of descending pain control pathways arising from the locus coeruleus and prefrontal cortex. This hypothesis is supported by evidence linking the ADRA2B gene to trigeminal nerve disorders, by recently published randomized controlled trial data demonstrating enhanced analgesia when guanfacine is added to lidocaine for trigeminal nerve blocks, and by the established analgesic activity of related α2-adrenergic agonists in neuropathic pain. However, it is important to note, direct evidence supporting these mechanisms in migraine treatment is lacking, and the proposed effects are largely based on biological plausibility derived from α2-adrenergic receptor pharmacology and experimental observations.

**Implications:**

Guanfacine has a favorable safety profile and a unique pharmacological signature compared with currently available migraine preventives. Despite indirect evidence presented here, it warrants evaluation in randomized, placebo-controlled trials for chronic migraine prevention.

## Introduction

Migraine is among the most disabling neurological disorders worldwide and is consistently ranked by the Global Burden of Disease Study as a leading cause of years lived with disability (1). The therapeutic landscape for migraine prevention has been transformed in the past decade by the introduction of monoclonal antibodies (mAbs) targeting calcitonin gene-related peptide (CGRP) or its receptor and by small-molecule CGRP receptor antagonists (gepants) (2–4). These agents have demonstrated efficacy, tolerability, and durability superior to most legacy oral preventives such as topiramate, propranolol, and amitriptyline (2, 5). Nevertheless, a substantial proportion of patients fail to achieve a clinically meaningful response to CGRP-targeting therapies. Even among initial responders, attack frequency tends to increase after treatment cessation, suggesting that the underlying disorder is regulated rather than reversed by these agents (6). Furthermore, the cost and route of administration of mAbs limit their accessibility globally, and oral preventives recommended in international guidelines remain associated with high discontinuation rates due to adverse events (4, 5).

Against this backdrop, drug repurposing—identification of new therapeutic indications for already-approved compounds—offers an attractive strategy to expand the migraine preventive armamentarium quickly and economically. Recent advances in Phenome-Wide Association Studies (PheWAS) coupling biobank-scale genetic data to electronic health records have made it possible to systematically generate hypothesis-driven candidates for repurposing (7). One such candidate is guanfacine, a selective α2A-adrenergic receptor (ADRA2A) agonist approved by the United States Food and Drug Administration for the treatment of hypertension in adults and for attention-deficit/hyperactivity disorder (ADHD) in children and adolescents (8). Unlike CGRP-targeting therapies, which primarily inhibit a single neuropeptide pathway, guanfacine may work by modulating several distinct pathways (outlined below). Moreover, its established safety profile, once daily dosing, and decades of clinical use make it an attractive candidate for drug repurposing.

In this hypothesis article, we propose that guanfacine may have a clinically meaningful preventive role in chronic migraine. The present hypothesis is distinguished by its foundation in genetics. Rather than arising solely from the known pharmacologic properties of guanfacine, it originated from a PheWAS association linking ADRA2B variants with trigeminal nerve disorders and is subsequently supported by converging evidence from trigeminal neuroanatomy, α2-adrenergic receptor pharmacology, locus coeruleus physiology, and emerging clinical data. Furthermore, despite isolated mentions, guanfacine is not recommended in current evidence-based migraine prevention guidelines, underscoring the absence of sufficient clinical evidence to support its routine use (9–11). Accordingly, the present work seeks to provide a comprehensive biologic framework to guide future preclinical investigation and prospective clinical trials.

The hypothesis is supported by three indirect but complimentary on three pillars: (1) PheWAS-derived genetic evidence implicating the ADRA2B subtype of the α2-adrenergic receptor family in trigeminal nerve disorders (12); (2) recently published randomized clinical trial data demonstrating that guanfacine, when added to lidocaine for trigeminal nerve blocks, enhances analgesia compared with lidocaine alone (12); and (3) a plausible neuropharmacological rationale linking α2-adrenergic receptor activation to inhibition of pro-nociceptive neurotransmitter release from trigeminal afferents, attenuation of peripheral and central sensitization, and modulation of descending pain-control pathways (13–15). These observations and studies do not establish the efficacy of guanfacine for migraine prevention, but rather provide a mechanistic framework supporting its biological plausibility and rationale for further investigation.

This article synthesizes these complementary lines of evidence into a unified mechanistic hypothesis, discusses the limitations of the existing clinical literature—including the single prior clinical trial of guanfacine for migraine prophylaxis (16)—and outlines a translational research strategy to guide future preclinical and prospective clinical studies.

## The hypothesis

We hypothesize that chronic oral treatment with guanfacine may reduce the frequency, severity, and duration of migraine attacks in patients with chronic migraine through three potentially complementary mechanisms operating at distinct levels of the trigeminovascular pain system. First, presynaptic α2-adrenergic receptor activation on trigeminal primary afferents may inhibit voltage-gated calcium influx and consequently suppress the release of CGRP, glutamate, substance P, and nitric oxide—neurotransmitters with potential to cause migraine pain and neurogenic inflammation. Second, sustained inhibition of nociceptive transmission at the trigeminocervical complex (TCC) could attenuate the peripheral and central sensitization processes that underlie migraine chronification, including cutaneous allodynia. Third, postsynaptic α2A-adrenergic receptor stimulation in the prefrontal cortex and brainstem nuclei (notably the locus coeruleus) augments endogenous descending pain inhibition, which may raise the threshold for migraine attack initiation.

## Pathophysiological framework

### The trigeminovascular system and migraine pain

The anatomical and physiological substrate of migraine pain is the trigeminovascular system, a network comprising primary afferent neurons of the trigeminal ganglion that project peripherally to the dura mater and large cerebral arteries and centrally to the TCC formed by the spinal trigeminal nucleus and upper cervical dorsal horn (13). From the TCC, second-order neurons relay nociceptive information to brainstem nuclei (notably the periaqueductal grey, parabrachial nucleus, and locus coeruleus), the hypothalamus, and the thalamus, from which third-order projections reach somatosensory, insular, and other cortical regions involved in the sensory, affective, autonomic, and cognitive dimensions of migraine (13).

During a migraine attack, peripheral trigeminal afferents become activated and sensitized through the release of pro-inflammatory and pain inducing mediators including CGRP, substance P, nitric oxide, and prostaglandin E2. CGRP is now widely regarded as a significant regulator of migraine pain: it is released into the cranial circulation during attacks, can provoke migraine-like attacks when infused intravenously in susceptible individuals, and is the molecular target of effective acute and preventive therapies (gepants and mAbs) (2, 13). CGRP signaling at the trigeminal ganglion, dura, and TCC mediates and modulates trigeminovascular nociceptive traffic; it sensitizes both Aδ meningeal nociceptors and second-order trigeminovascular neurons of the TCC, ultimately contributing to cephalic allodynia, scalp tenderness, and the sequential symptomatic features of an attack (13, 14).

Glutamate is the principal excitatory neurotransmitter co-released with CGRP from trigeminal ganglion neurons (17), and elevated cortical glutamatergic tone is a feature of the interictal migraine brain (13). Repeated activation of these peripheral and central pathways is thought to drive the transformation from episodic to chronic migraine through neuroplastic changes—peripheral sensitization, central sensitization, and dysfunction of descending pain modulation—that together lower the threshold for attack initiation and amplify pain transmission (14, 15).

Although CGRP has emerged as a primary therapeutic target in migraines, other neuropeptides also contribute to trigeminovascular signaling. For example, substance P has been found to play a role in neurogenic inflammation and nociceptive pathways within the TCC. Additionally pituitary adenylate cyclase-activating polypeptide (PACAP) has emerged as another potential mediator in migraine physiology. PACAP-38 has been shown to be coreleased with CGRP from trigeminal afferents and is widely present throughout the trigeminovascular system (18). Intravenous PACAP-38 infusions have also been shown to prove migraine-like phenomena in individuals. Through studying clonidine, α2-adrenergic receptor targeting has been shown to decrease substance P from dorsal root ganglion neurons (19). Evidence regarding α2-adrenergic signaling on PACAP remains limited (20). Whether guanfacine similarly modulates substance P or PACAP signaling within the trigeminovascular system remains unknown and warrants further investigation.

### Central sensitization, allodynia, and migraine chronification

Central sensitization clinically manifests as cutaneous allodynia, which is associated with headache-related disability and chronic migraine. Cutaneous allodynia is reported by up to two-thirds of patients with migraine, occurs both ictally and interictally in chronic migraine, and predicts headache-related disability and the development of chronic migraine (14, 15).

Calcitonin gene-related peptide plays a central role in this process. Preclinical studies show that CGRP released in the dura, trigeminal ganglion, and brainstem promotes the activation of satellite glial cells, the release of nitric oxide and pro-inflammatory cytokines, and the sustained sensitization of peripheral and central trigeminovascular neurons (21). CGRP mAbs prevent the development of activity-induced sensitization of dural-projecting nociceptors and of wide-dynamic-range neurons in the TCC (13). Critically, however, central sensitization can persist even after CGRP-pathway blockade in a substantial subset of patients, indicating that complementary therapeutic targets are required to fully reverse the chronification cascade.

### Descending pain modulation and the locus coeruleus

Descending pain modulatory systems originating in the periaqueductal grey, rostral ventromedial medulla, locus coeruleus, and prefrontal cortex can lead to inhibitory modulation over spinal and trigeminal nociceptive transmission (22). Loss of descending inhibition is increasingly recognized as a permissive factor in the chronification of pain disorders (22). The locus coeruleus, the principal source of brain norepinephrine, projects diffusely throughout the central nervous system and exerts complex, bidirectional influences on migraine.

Classically, ascending projections are thought to modulate cortical excitability and susceptibility to cortical spreading depression, while descending noradrenergic projections are thought to inhibit nociceptive transmission at the spinal cord and TCC (23). Neuromelanin-sensitive magnetic resonance imaging suggests altered locus coeruleus signal in patients with migraine with aura (24), and preclinical lesioning studies indicate that locus coeruleus disruption enhances cortical spreading depression susceptibility while inhibiting dural-evoked TCC activation (23). Together, these data implicate the noradrenergic system as a key node in migraine pathophysiology and provide a rationale for therapies that modulate α2-adrenergic signaling.

However, it is important to recognize that although the descending pathways are thought of as pain-relieving, more recent literature has shown that descending pathways can also have a pronociceptive effect. Optogenetic studies have shown that certain neuronal subpopulations in the locus coeruleus drive thermal nociception (25). Moreover, more recent studies have challenged the notion that the locus coeruleus is responsible for driving diffuse noxious inhibitory control (DNIC). A study by Kucharczyk et al. demonstrated that the primary driver of the DNIC was A5 neurons rather than the locus coeruleus. It was proposed, although not confirmed, that overactivation of the locus coeruleus may lead to suppression of A5 neurons which in turn increased pain signals (26). The role of pain modulation of this effect is still currently being investigated. As such, the overall pharmacological effect of descending pathways on the locus coeruleus activity may be context driven. It may be more complex than originally thought; however additional studies are required to fully elucidate the descending pain pathways and identify which neuronal subpopulations within the locus coeruleus are responsible for driving pain relief.

## α2-adrenergic receptor pharmacology and the case for guanfacine

### Molecular mechanisms of α2-adrenergic analgesia

The α2-adrenergic receptor (ADRA2) family comprises three highly homologous G-protein-coupled receptor subtypes—α2A, α2B, and α2C—each encoded by distinct genes (ADRA2A, ADRA2B, ADRA2C) and characterized by overlapping but distinct anatomical distributions and functions (8, 27). All three subtypes signal predominantly through inhibitory Gi/Go proteins to suppress adenylate cyclase activity, decrease intracellular cyclic AMP, inhibit voltage-gated calcium channels, and activate G-protein-coupled inwardly rectifying potassium channels. The resulting presynaptic effect leads to a reduction in calcium-dependent neurotransmitter release (27).

In the somatosensory system, presynaptic α2-adrenergic receptors expressed on primary afferent terminals are a major substrate of endogenous noradrenergic analgesia (27, 28). Activation of these receptors has been reported to reduce calcium-dependent release of glutamate, CGRP, substance P, and other pro-nociceptive neurotransmitters into the spinal and medullary dorsal horn. Whether guanfacine produces comparable effects on CGRP, glutamate, or substance P has not been formally demonstrated. The clinical analgesic efficacy of intrathecal and epidural clonidine, the perioperative use of dexmedetomidine, and the analgesic actions of α2-adrenergic agonists in models of neuropathic pain are thought to involve this mechanism (27, 29). Some preclinical studies suggest that α2A receptor activation reduces neuropathic pain behaviors, including mechanical and thermal allodynia (29), and that α2-adrenergic agonists potentiate the local anesthetic action of lidocaine (28).

### ADRA2B expression in the trigeminal ganglion

Of particular relevance to migraine, the ADRA2B gene is expressed in the human trigeminal ganglion, and α2B and α2C subtype mRNAs predominate in human dorsal root ganglion neurons (30). This expression pattern positions α2-adrenergic receptors as a potential pharmacological target on the neurons whose pathological hyperexcitability is thought to contribute to migraine pain. By engaging presynaptic α2-adrenergic receptors on trigeminal afferents, an agonist such as guanfacine could in principle reduce the release of CGRP and other algogenic mediators in the dura, trigeminal ganglion, and TCC—which could produce an antimigraine effect that is mechanistically complementary to, but distinct from, CGRP-pathway blockade.

### Guanfacine: pharmacology, safety, and differentiating features

Guanfacine is a centrally acting selective α2A-adrenergic receptor agonist with approximately 15- to 20-fold higher selectivity for the α2A subtype than clonidine, the prototypical α2-adrenergic agonist (8, 31). Its oral bioavailability is approximately 80%, and its half-life of 17 h permits once-daily dosing (8). Guanfacine is approved at doses up to 4 mg per day for hypertension in adults and up to 7 mg per day (extended-release) for ADHD in children and adolescents (8). Beyond its labelled indications, guanfacine is widely used off-label for tic disorders, generalized anxiety disorder, post-traumatic stress disorder, opioid-induced hyperalgesia, and substance use disorders, reflecting a remarkable breadth of central nervous system activity (31).

Compared with other α2-adrenergic agonists, guanfacine has several pharmacological features that make it particularly attractive as a candidate migraine preventive. First, its higher α2A selectivity may contribute to reduced sedation, lower incidence of hypotension, and a lower risk for rebound hypertension upon discontinuation compared with clonidine (31, 32). Second, its long half-life supports stable plasma concentrations and once-daily dosing, an important determinant of long-term adherence in chronic preventive therapy. Third, guanfacine has a well-characterized safety profile from decades of use, with the most common adverse events being mild and transient: somnolence, fatigue, headache, dry mouth, dizziness, and modest decreases in heart rate and blood pressure (32). Fourth, guanfacine has no vasoconstrictor activity and therefore would not be contraindicated in patients with cardiovascular comorbidities for whom triptans and ergot derivatives are problematic (1).

In addition to direct effects on trigeminal afferents, guanfacine acts on postsynaptic α2A receptors on prefrontal cortex pyramidal neurons, where it strengthens working memory networks by inhibiting cAMP–HCN channel signaling (31). This prefrontal action has been associated with top-down cognitive control of attention and emotion and has been postulated to reinforce descending pain inhibition (31, 33). Guanfacine has also been shown to inhibit interictal epileptiform activity and sodium currents in prefrontal cortex pyramidal neurons, suggesting potential network-stabilizing effects relevant to disorders of cortical excitability such as migraine (34).

## Supporting evidence for the hypothesis

### PheWAS identification of ADRA2B as a trigeminal pain target

The genetic case for α2-adrenergic targeting in trigeminal pain emerged from a PheWAS analysis performed in the BioVU biobank at Vanderbilt University Medical Center, which links genotype data from patients to longitudinal de-identified electronic health record phenotypes (12). The missense single-nucleotide variant rs29000569 in the ADRA2B gene showed an association with the Phecode for trigeminal nerve disorders (170 cases, 25,739 controls; odds ratio 4.62, *p* = 0.003075) (12). This association was the genetic foundation for a subsequent drug-repurposing program identifying guanfacine as a candidate analgesic for trigeminal pain. Although the article does not describe the *p* value after correction for phenome wide testing, there remains an association between the ADRA2B gene and trigeminal nerve disorders.

PheWAS approaches have a strong track record of replicating known gene-disease associations and of generating drug-repurposing hypotheses that translate successfully into clinical studies (7). The convergence of PheWAS-derived genetic evidence with established pharmacology and anatomy—ADRA2B receptors on trigeminal ganglion neurons inhibiting calcium-dependent neurotransmitter release—provides preliminary human genetic support, that when considered alongside pharmacological properties supports further investigation into the use of guanfacine in migraine pain.

### Randomized trial evidence: guanfacine as a trigeminal nerve block adjuvant

The first prospective, randomized, double-blind clinical trial of guanfacine for trigeminal pain was recently published (12). In this proof-of-concept, single-center crossover trial, 37 patients with trigeminal neuralgia or painful trigeminal neuropathy were randomized to receive trigeminal nerve blocks of the V2 and V3 branches with lidocaine alone or lidocaine plus 250 μg guanfacine. Numerical Rating Scale (NRS) pain scores over the first 8 h after injection were significantly lower in the guanfacine-plus-lidocaine arm than in the lidocaine-alone arm (*p* < 0.001), reaching a maximum difference of 1.02 NRS units (95% confidence interval 0.70–1.33) at approximately 4.5 h after injection (12). The combination was well tolerated, with no serious adverse events and no statistically significant difference in adverse-event rates between arms (12). It is important to note that the 1.02 NRS difference falls below 2.0 which is the commonly used benchmark for minimal clinically important difference for the NRS (35). As such, the clinical significance of these findings should be interpreted with caution. Additionally, because this study evaluated locally administered guanfacine as an adjuvant to trigeminal nerve blocks in patients with trigeminal neuralgia, these results cannot be directly extrapolated to chronic systemic administration for migraine prevention. Rather, several aspects of its results provide support for the biological mechanism of our present hypothesis. First, the dose used (250 μg) was substantially below the oral doses used for hypertension and ADHD (8), yet produced a modest but statistically significant analgesic effect. Second, the analgesic action of guanfacine in this trial is consistent with the proposed mechanism, although the exact pharmacological pathways still remain incompletely understood. The hypothesized mechanism involves presynaptic inhibition of neurotransmitter release from trigeminal afferents and possible diffusion of the drug to the trigeminal ganglion through the foramen rotundum and ovale (12). Third, the trial demonstrates that the combination of guanfacine with a sodium-channel blocker (lidocaine) may have produced additive or synergistic analgesia, consistent with engagement of two distinct, complementary mechanisms for inhibiting trigeminal nociceptive transmission.

While these findings provide mechanistic support for our hypothesis, important distinctions remain between trigeminal neuralgia and migraine pathophysiology. Trigeminal neuralgia is classically seen with neurovascular compression leading to demyelination, axonal hyperexcitability, and ectopic firing within the trigeminal nerve (36). However, painful trigeminal neuropathy has a more complex and less well understood pain process. On the other hand, migraine represents a complex neurovascular disorder with many distinct mechanisms including activation of the TCC, CGRP mediated inflammation, and cortical hyperexcitability (37). Despite these important differences, both disorders engage trigeminal nociceptive pathways, which provide a shared anatomical target through which adrenergic signaling may influence pain processing (38).

The nerve-block trial should not be interpreted as evidence that the acute effects of locally administered guanfacine will translate to chronic oral migraine prevention. Rather, the relevance of this trial is that it demonstrates that guanfacine can modulate nociceptive transmission within the trigeminal system. Additional evidence provides a biological rationale for why alpha-2 adrenergic modulation may also be relevant with systemic administration.

First, the trigeminal ganglion lies outside the blood–brain barrier, making it accessible to systemic agents and therefore providing a potential target for oral medications (39). Prior studies have shown that alpha-2 receptors can directly modulate neuronal excitability within the trigeminal ganglion. Activation of alpha-2 receptors has been shown to hyperpolarize neurons, suppress recurrent firing, and inhibit voltage-gated sodium channels (40, 41). In addition to the trigeminal ganglion, alpha-2 receptors can also exert antinociceptive effects at spinal and supraspinal sites, potentially modulating central sensitization which is known to play an important role in chronic migraine pathophysiology (42). Together, these findings provide a biological rationale for evaluating chronic systemic guanfacine in migraine. However, whether the acute effects observed with local administration translate to preventive efficacy with chronic oral dosing requires detailed investigation.

### α2-adrenergic agonists in neuropathic and postoperative pain

The broader pharmacological literature supports the analgesic activity of α2-adrenergic agonists in distinct conditions sharing some pathophysiological features with chronic migraine, including peripheral and central sensitization, allodynia, and neuropathic components. Dexmedetomidine, a highly selective α2 agonist, attenuates neuropathic pain in rodent models and is used clinically for postoperative analgesia and for refractory neuropathic pain crises (27, 43). Clonidine has shown efficacy in diabetic neuropathy and as an adjuvant for regional anesthesia (27, 28). Tizanidine, a structurally related α2 agonist with about one-tenth the antihypertensive potency of clonidine, has been used for headache prophylaxis with reported benefit (44).

Guanfacine, with its higher α2A selectivity and longer half-life, has been investigated specifically for opioid-induced hyperalgesia—a condition characterized by central sensitization and lowered nociceptive thresholds—through an ongoing US-based clinical trial (45). Animal studies have demonstrated that intrathecal guanfacine produces antinociception with a longer duration than clonidine and without evidence of spinal or nerve-root toxicity at therapeutic doses (28). These data, while indirect, support both the plausibility and the clinical safety of administering guanfacine for sustained periods to modulate trigeminal sensitization in chronic migraine.

### Reappraising the single prior trial of guanfacine and trials of clonidine and tizanidine in migraine prevention

A single placebo-controlled randomized trial of guanfacine in migraine prevention was conducted in the late 1980s and reported only as a 536-word extended abstract from the International Headache Congress in 1989 (16). In this small two-site parallel-group trial, guanfacine at 1 mg daily appeared to significantly reduce migraine frequency compared with placebo (*p* = 0.0053), though only *p* values were reported and full results from the second study site were never published (16). The AHS/AAN evidence-based guideline for migraine prevention nonetheless classified guanfacine as Level C (possibly effective) based on this study (16), a classification that was subsequently challenged in a critical commentary highlighting the methodological limitations of relying on an unpublished, incompletely reported trial (16).

One should not consider this single trial presented only as an abstract to constitute conclusive evidence of guanfacine’s efficacy in migraine prevention. Rather, one should view its result as hypothesis-generating and broadly consistent with the mechanistic framework articulated here. Importantly, the trial was performed with the immediate-release formulation prior to the contemporary understanding of CGRP and central sensitization in migraine, used only a single fixed dose, and did not specifically enroll patients with chronic migraine. A contemporary, adequately powered trial—employing extended-release guanfacine, endpoints (monthly migraine days, 50% responder rates, headache-related disability scales), and enriched enrollment of patients with chronic migraine and incomplete response to standard preventives—would provide definitive evidence.

It is important to consider that prior α2-adrenergic agonists, specifically clonidine, have been less successful in migraine prevention than expected. The largely negative trials raise the question whether guanfacine would fare differently as they both have a similar mechanism of action. Multiple double blind placebo trials did not show any prophylactic benefit for clonidine at doses of 50 and 150 μg. However, the failure of clonidine for migraine prevention should not imply that guanfacine will fare a similar result. Guanfacine has different pharmacodynamic and pharmacokinetic properties which could yield differing results. Compared to clonidine, guanfacine has a longer half-life and has a greater α2A-receptor selectivity (46, 47). Additionally, head-to-head studies between clonidine and guanfacine have shown that guanfacine produced less somnolence and dry mouth than clonidine, which may allow for dose escalation to levels sufficient for trigeminal α2-receptor modulation. The prior clonidine trials may have used doses (50–150 μg daily) that could have been too low to produce an analgesic effect. That said, although these differences between the two drugs exist, it is not clear if guanfacine will necessarily be superior to clonidine.

The role of alpha-2 adrenergic modulation in headache treatment comes, in part, from tizanidine. Prior data from a double-blind, placebo-controlled, multicenter trial of patients with chronic daily headache, found that tizanidine was superior to placebo across several headache measures (44). Importantly, the efficacy of tizanidine, a less selective alpha-2 adrenergic agonist, shows that alpha-2 receptor selectivity alone is unlikely to determine efficacy in headache prevention. However, these findings provide additional clinical support for the broader hypothesis that alpha-2 adrenergic modulation may have therapeutic relevance in migraine pathophysiology. In order to see a therapeutic effect with tizanidine, relatively high doses were required for the trial. This limited overall tolerability, as some patients experienced somnolence, dizziness, and dry mouth (44). Thus, guanfacine’s pharmacokinetic properties, including its longer half-life and greater alpha-2A selectivity, may be beneficial over less selective alpha-2 agonists. However, it is important to acknowledge that these findings do not imply that guanfacine may be more effective for migraines, but provide a rationale for evaluating guanfacine despite mixed clinical experiences with other alpha-2 agonists.

## Clinical implications and translational strategy

### Potential patient populations most likely to benefit

The mechanistic framework outlined here supports a biologically plausible mechanism whereby guanfacine may be particularly effective in subgroups of patients with chronic migraine in whom α2-adrenergic mechanisms are most likely to be engaged. First, patients with prominent cutaneous allodynia and other markers of central sensitization—who often respond suboptimally to acute therapies and represent a high-disability subset—would be the theoretical responders, given the well-established role of α2A receptor signaling in reversing central sensitization in preclinical pain models (14, 15, 27). Second, patients with comorbid anxiety, insomnia, post-traumatic stress disorder, or ADHD—conditions that are themselves indications or off-label uses for guanfacine and that are commonly comorbid with migraine (1, 48)—may obtain dual benefit from a single intervention. Third, patients in whom triptans, gepants, or CGRP mAbs are contraindicated, ineffective, or poorly tolerated would represent a population of substantial unmet need.

Conversely, patients with baseline hypotension, bradycardia, or syncope, or those on concurrent antihypertensives, would require careful selection and monitoring. The cardiovascular profile of guanfacine—modest dose-dependent reductions in heart rate and blood pressure—is generally well-tolerated but warrants standard precautions, particularly in older patients and those with autonomic dysregulation (32). Additionally, side effects including sedation, lethargy and fatigue may limit the tolerability of its use in some patients with migraines (49).

As guanfacine has not yet been evaluated for migraine prevention, its clinical role remains uncertain. However, the most plausible application would be as an adjunctive or alternative treatment for patients with inadequate response to or who are unable to tolerate current first line migraine therapies.

### Proposed mechanism of action in chronic migraine

We suggest that chronic oral guanfacine acts in chronic migraine through three possible integrated mechanisms. At the peripheral level, sustained α2-adrenergic receptor activation on trigeminal primary afferents may inhibit calcium-dependent release of CGRP, glutamate, substance P, and nitric oxide into the dura and TCC. This could reduce neurogenic inflammation, attenuate peripheral sensitization of meningeal nociceptors, and decreases ascending nociceptive traffic. At the level of the trigeminal ganglion and TCC, α2-adrenergic receptor activation reduces both basal and stimulated neurotransmitter release and may attenuate central sensitization that drives allodynia, hyperalgesia, and the migraine-chronification trajectory. At the brainstem and cortical level, postsynaptic α2A receptor stimulation may augment descending noradrenergic inhibition and strengthen prefrontal top-down cognitive and emotional regulation, potentially raising the threshold for attack initiation in response to internal and external triggers.

This three-tiered mechanism is mechanistically complementary to that of CGRP-pathway antagonists, which act primarily at peripheral and possibly trigeminal ganglion sites (2), and to that of onabotulinumtoxinA, which inhibits release of inflammatory neuropeptides from trigeminal afferents but does not address descending pain modulation. The combination of guanfacine with a CGRP mAb or gepant is therefore biologically plausible and could be examined in patients with incomplete response to single-mechanism therapy.

### A proposed research agenda

Several research steps would be required to rigorously test the hypothesis. First, preclinical studies should characterize the effect of systemic guanfacine on stimulated CGRP release from trigeminal afferents, on neurogenic dural inflammation, on the development and reversal of cutaneous allodynia and on cortical spreading depression susceptibility in established animal models of chronic migraine. These studies should evaluate dose–response relationships and explore additive or synergistic interactions with CGRP-pathway antagonists.

Second, a Phase II randomized, double-blind, placebo-controlled trial of extended-release guanfacine in adults with chronic migraine should be undertaken, using current International Headache Society recommendations for trial endpoints (3). Primary endpoints could be the change from baseline in monthly migraine days at 12 weeks and the proportion of participants achieving a ≥ 50% reduction in monthly migraine days. Secondary endpoints should include the impact on cutaneous allodynia, acute medication use, headache-related disability (e.g., Migraine Disability Assessment scale), and quality of life.

Pre-specified subgroup analyses should evaluate efficacy in patients with prior CGRP mAb failure, in those with prominent allodynia, and in those with psychiatric and sleep-related comorbidities. Exploratory subgroup analysis could include cutaneous allodynia scores and quantitative sensory testing. In patients with prior CGRP failure, looking into plasma CGRP concentrations may provide additional insight.

Third, depending on Phase II results, an active-comparator Phase III trial against an established oral preventive (e.g., topiramate) and/or a combination trial with a CGRP mAb in non-responders would establish the role of guanfacine in the broader preventive armamentarium. Pharmacogenomic substudies could examine whether the ADRA2B rs29000569 variant (12) or related polymorphisms predict response, advancing the goal of precision medicine in headache disorders. Finally, further investigation with functional MRIs to assess alternation in pain pathways and Neuromelanin sensitive MRIs to evaluate locus ceruleus activity may also be of utility in future analysis.

## Limitations of the hypothesis

Several limitations must be acknowledged. First, direct clinical evidence of guanfacine efficacy in migraine is limited to a single small trial from 1989 with incomplete reporting (16), and the recently completed trigeminal nerve block trial—while supportive of the underlying pharmacology—examined a different patient population and route of administration (12). Whether the analgesic effects observed in trigeminal neuralgia translate to chronic migraine remains to be demonstrated.

Second, the precise contribution of each α2-adrenergic receptor subtype (α2A, α2B, α2C) to any putative antimigraine effect is uncertain. Guanfacine is selective for α2A, but the PheWAS signal driving the present hypothesis arose from variants in ADRA2B (12), and human dorsal root ganglion neurons express predominantly α2B and α2C subtype mRNAs (30). Whether α2A selectivity is optimal for trigeminal targeting, or whether a less subtype-selective agent (such as clonidine) might offer superior efficacy, requires investigation. Although these receptor subtypes may differ in their tissue distribution, all three are Gi/Go-coupled G-protein-coupled receptors that share several downstream signaling mechanisms and have functional redundancy and cross-talk. Additionally, prior research has shown the downstream effects of each of these receptor subtypes have shared many similarities including modulation of ion changes and adenylate cyclase (50). As such, although PheWAS specifically identified ADRA2B, this signal could implicate the broader α2-adrenergic signaling pathway, rather than only that receptor subtype. However, before a more definitive conclusion can be drawn, the translational relevance of receptor subtype distribution and function derived from animal models needs validation in human biology.

Third, dose, formulation, and timing for migraine prevention have not been established. The doses used for ADHD (1–7 mg daily of extended-release) and hypertension (1–4 mg daily of immediate-release) may not be optimal for engaging trigeminal targets, and pharmacokinetic and pharmacodynamic studies will be needed to define the appropriate therapeutic window.

Fourth, the safety of long-term daily guanfacine in adult migraine populations requires confirmation. While decades of cardiovascular use and more recent ADHD experience suggest a favorable safety profile (8, 32), migraine populations are predominantly women of reproductive age. The FDA label for guanfacine states that data over decades of use have not identified a drug associated risk with major birth defects or adverse maternal or fetal outcomes. However, the FDA acknowledges that the use of guanfacine in pregnant women has been infrequent (51). Pregnancy considerations must be addressed in future human studies.

Additionally, there has yet to be an experimental study demonstrating guanfacine-mediated inhibition of CGRP, glutamate, substance P, and nitric oxide release from trigeminal afferents. The present hypothesis largely stems from established studies on α2-adrenergic receptor physiology. As such, we propose a biological plausibility mechanism in this study, but direct studies looking at the effects of guanfacine on the above substrates are required for validation.

Finally, while we have emphasized the unique pharmacological profile of guanfacine, the broader class of α2-adrenergic agonists has been less successful in migraine prevention than initial enthusiasm anticipated. Clonidine, an older and less selective α2 agonist, has been studied in migraine without convincing efficacy (1, 4). It is possible that the molecular features distinguishing guanfacine from clonidine—greater α2A selectivity, longer half-life, less sedation, better tolerability—are also clinically relevant for migraine prevention, but this remains to be demonstrated.

## Conclusion

Chronic migraine remains a major source of disability worldwide, and despite recent advances a substantial fraction of patients do not benefit adequately from currently available preventive therapies. Drawing on PheWAS-derived genetic evidence linking ADRA2B to trigeminal nerve disorders, recently published randomized controlled trial data showing enhanced analgesia when guanfacine is added to lidocaine for trigeminal nerve blocks, and a rich mechanistic literature on α2-adrenergic receptor signaling, we have proposed that guanfacine warrants further consideration as a preventive medication for migraines. Its proposed mechanism of action—presynaptic inhibition of pro-nociceptive neurotransmitter release from trigeminal afferents, attenuation of peripheral and central sensitization, and modulation of descending pain inhibition—is mechanistically complementary to existing therapies, and its established safety profile, long half-life, and lack of vasoconstrictor activity support investigation for chronic preventive use. Collectively, these observations call for additional preclinical studies and randomized controlled trials while illustrating the potential of integrating genetic evidence with mechanistic and clinical evidence to guide hypothesis-driven drug repurposing.
